# *Mycobacterium tuberculosis* Rv2617c is involved in stress response and phage infection resistance

**DOI:** 10.1016/j.heliyon.2024.e27400

**Published:** 2024-03-05

**Authors:** Liadrine Moukendza Koundi, Ulrich Aymard Ekomi Moure, Funmilayo Grâce Boni, Insaf Hamdi, Lin Fan, Jianping Xie

**Affiliations:** a Institute of Modern Biopharmaceuticals, State Key Laboratory Breeding Base of Eco-Environment and Bio-Resource of the Three Gorges Area, Key Laboratory of Eco-environments in Three Gorges Reservoir Region, Ministry of Education, School of Life Sciences, Southwest University, Chongqing, China; b The Ninth People's Hospital of Chongqing, Affiliated Hospital of Southwest University, Chongqing, China; c Cancer Center, Medical Research Institute, Southwest University, 400716, Chongqing, China; d Shanghai Clinic and Research Center of Tuberculosis, Shanghai Pulmonary Hospital, Tongji University School of Medicine, Shanghai Key Laboratory of Tuberculosis, Shanghai, China

**Keywords:** *Mycobacterium tuberculosis*, Rv2617c, DoxX domain, Stress responses, Virulence, Mycobacteriophage resistance

## Abstract

*Mycobacterium tuberculosis* (*M. tuberculosis)* is the pathogen of human tuberculosis (TB). Resistance to numerous *in vivo* stresses, including oxidative stress, is determinant for *M. tuberculosis* intracellular survival, and understanding associated mechanisms is crucial for developing new therapeutic strategies. *M. tuberculosis* Rv2617c has been associated with oxidative stress response when interacting with other proteins in *M. tuberculosis*; however, its functional promiscuity and underlying molecular mechanisms remain elusive. In this study, we investigated the phenotypic changes of *Mycobacterium smegmatis* (*M. smegmatis*) expressing Rv2617c (Ms_Rv2617c) and its behavior in the presence of various *in vitro* stresses and phage infections. We found that Rv2617c conferred resistance to SDS and diamide while sensitizing *M. smegmatis* to oxidative stress (H_2_O_2_) and altered mycobacterial phenotypic properties (single-cell clone and motility), suggestive of reprogrammed mycobacterial cell wall lipid contents exemplified by increased cell wall permeability. Interestingly, we also found that Rv2617c promoted *M. smegmatis* resistance to infection by phages (SWU1, SWU2, D29, and TM4) and kept phage TM4 from destroying mycobacterial biofilms. Our findings provide new insights into the role of Rv2617c in resistance to oxide and acid stresses and report for the first time on its role in phage resistance in *Mycobacterium.*

## 1 Introduction

*Mycobacterium tuberculosis* (*M. tuberculosis*) is the pathogen of tuberculosis (TB), a major public health threat worldwide. *M. tuberculosis* can persist and evade the host immune response, leading to a chronic infection that can last for decades. The survival of *M. tuberculosis* in the host requires the bacterium to resist various stresses, including oxidative stress [[1], [2], [3]]. Oxidative stress is a major challenge for *M. tuberculosis*, as it leads to the production of reactive oxygen species (ROS), which can damage bacterial cellular components and disrupt cellular processes [4]. However, *M. tuberculosis* has evolved several mechanisms for detoxifying ROS and maintaining cellular homeostasis to combat oxidative stress. These mechanisms include the production of antioxidants and the regulation of enzymes involved in the ROS detoxification pathway.

Mycobrowser (mycobrowser.epfl.ch) lists over 4000 open reading frames (ORFs) in the *M. tuberculosis* genome. However, around 1803 gene-producing proteins are still unclassified, and 68 are potential transmembrane proteins. Rv2617c, found in mycobacterium species (including *M. tuberculosis*), belongs as well to that category of proteins and has been implicated in oxidative stress response in *M. tuberculosis*. Although some studies have suggested that Rv2617c may play a role in oxidative stress response [5], the study of Rv2617c in *M. tuberculosis* is still in its early stages, and much remains to be done to determine its function and significance in this bacterium, especially the phenotypes and molecular mechanisms by which it confers resistance not only to oxidative stress and other harsh conditions encountered by *M. tuberculosis* within macrophages but also to mycobacteriophage infections.

Discovered more than 100 years ago by Twort and d'Herelle, phages (or bacteriophages) are viruses that infect and replicate within bacteria through lytic or lysogenic lifestyles [6]. Originally used as a tool in clinical treatments against pathogenic bacteria, they now emerge as potential alternative treatments to tackle mycobacterial drug resistance, including multiple drug-resistant (MDR)-TB and extensively drug-resistant (XDR)-TB infections, offering the possibility of shortening antibiotic regimens and avoiding treatment failures [7]. Mycobacteriophages like SWU1, SWU2, D29, L5, and TM4 can infect mycobacteria [[8], [9], [10], [11], [12]]. However, intracellular mycobacteria like *M. tuberculosis* possess an arsenal of defense that promotes their resistance to phage infections. Therefore, identifying *M. tuberculosis* effectors involved in phage infection resistance and underlying molecular mechanisms would provide more insights into the molecular pathogenicity and allow the discovery of potential targets for phage-based therapy.

By overexpressing Rv2617c in *M. smegmatis*, a non-pathogenic mycobacterial strain and surrogate of *M. tuberculosis*, we found that Rv2617c is relatively involved in mycobacterial stress responses and alters mycobacterial phenotypic properties probably through cell wall content alteration associated with an increased cell wall permeability. Besides, Rv2617c confers *M. smegmatis* resistance to mycobacteriophage infections and promotes biofilm formation in the presence of TM4 mycobacteriophage. To our knowledge, this study is the first to report on Rv2617c involvement in resistance to mycobacteriophage infections.

## 2 Results

### 2.1 Rv2617c belongs to the DoxX family proteins

**Fig. 1:**
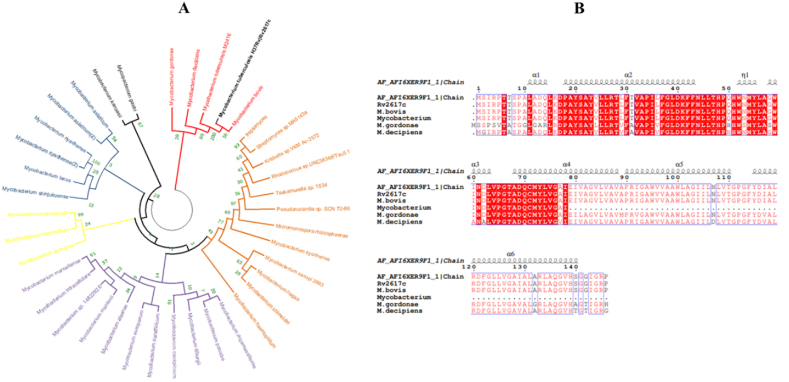
Rv2617c protein is highly conserved among mycobacteria. (A) A phylogenetic tree was obtained after blasting the *M. tuberculosis* Rv2617c protein sequence in NCBI. *M. tuberculosis* H37Rv Rv2617c is closer to its orthologs in *M. bovis*, *M. tuberculosis* 1418, *M. decipiens*, and *M. gordonae*. (B) Multiple sequence alignment (MSA) of *M. tuberculosis* Rv2617c protein with its orthologs from *M. bovis*, *M. tuberculosis* 1418, *M. decipiens*, and *M. gordonae*. The conserved DoxX domain is shaded in red, and the AF_AFI6XER9F1_Chain1 is the predicted secondary structure model of the probable transmembrane protein (Rv2617c) from *M. tuberculosis* DoxX family proteins (https://www.rcsb.org/structure/AF_AFI6XER9F1).

The retrieved Rv2617c protein sequence from Mycobrowser was blasted in the National Center for Biotechnology Information (NCBI) using the command Blastp (blast proteins). Mycobacterial homologous proteins of Rv2617c were then used to build a phylogenetic tree (Fig. 1A). Then, the four mycobacterial strains closer to *M. tuberculosis* H37Rv harboring the Rv2617c protein were aligned together with Esprit 3.0 (https://espript.ibcp.fr/ESPript/ESPript/index.php) to determine the leaflet composition and the shared domains. We found that the Rv2617c protein is made of six alpha (α) and one êta (η) leaflets organized as the following α_1_, η_1_, α_2_, α_3_, α_4_, α_5_, and α_6_, and shares a highly conserved DoxX domain (Fig. 1B). In contrast to the DoxX domain found in *archaea* sulfur oxidation proteins carried by the integral membrane protein DoxX (Rv3005c), the DoxX domain from the Rv2617c protein is proposed to form a membrane-associated oxidoreductase complex (MRC) together with the superoxide-detoxifying enzyme (SodA) and the predicted thiol-oxidoreductase (SseA), and DoxX impairment may lead to defective recycling of mycothiol and accumulation of cellular oxidative damage [13].

### 2.2 Rv2617c is heterologously expressed in *M. smegmatis*

**Fig. 2:**
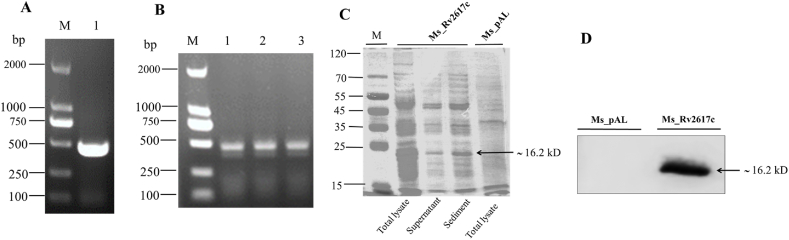
Heterologous expression of *M. tuberculosis* Rv2617c in *M. smegmatis*. (A) PCR amplification of Rv2617c from *M. tuberculosis* H37Rv genome. (B) The constructed Ms_Rv2617c strain was cultured in a 7H9 medium supplemented with acetamide and then subjected to PCR amplification to detect the Rv2617c gene with specific primers. (C) The cell lysates prepared from acetamide-induced Ms_pAL and Ms_Rv2617c cultures were analyzed by SDS-PAGE and visualized under UV after Coomassie Brilliant Blue staining to identify the His-Tag-associated Rv2617c protein. (D) Western Blot analysis of cell wall lysates of both strains. The His-tag-associated Rv2617c protein was targeted with anti-His-tag antibodies. See original and uncropped images in supplementary material.

*M. smegmatis* mc^2^ 155 is a fast-growing mycobacterium frequently used as a surrogate to study the functional and molecular mechanisms of *M. tuberculosis* genes. Since *M. tuberculosis* Rv2617c gene has no orthologs in *M. smegmatis*, no knockout was required. Thus, Rv2617c was PCR-amplified with specific primers (Table 1) from *M. tuberculosis* H37Rv genome to get the expected size of 441 bp (Fig. 2A); the latter amplicon was then ligated to the plasmid pALACE (with His-tag) and the whole inserted into competent *M. smegmatis* strains to get the recombinant strain Ms_Rv2617c overexpressing the Rv2617c protein (Fig. 2B). The Ms_pAL strain containing only the plasmid pALACE-(with His-tag) served as the control. Thereafter, Ms_Rv2617c and Ms_pAL total proteins were collected after acetamide induction for 12 h of both strain cultures and analyzed. The expected 16.2 kDa (including the 0.8 kDa of His-tag) recombinant protein can be detected by both SDS-PAGE following Coomassie brilliant blue staining (Fig. 2C) and Western Blot with anti-His-tag antibody, while in Ms_pAL strain no band was detected in the whole cell lysate (Fig. 2D). Altogether, the above findings indicate the successful Rv2617c overexpression in *M. smegmatis*.

**Table 1:** Strains, plasmids, and primers used in this study.

Strain	Description	Source
*M. smegmatis*	Wild type *M. smegmatis* mc^2^155 strain	Invitrogen
Ms_pAL	M. smegmatis strain transformed with vector pALACE	This study
Ms_Rv2617c	*M. smegmatis* strain transformed with vector pALACE-Rv2617c	This study
*E. coli* DH5α	The strain used for plasmid proliferation	Invitrogen
**Plasmid**	**Description**	**Source**
pALACE	A replicative plasmid with His-tag was used for gene expression in *M. smegmatis*, conferring hygromycin (Hyg) resistance.	Invitrogen
**Primer**	**Sequence (5′- 3′)**	**Source**
pAL-Rv2617c-F	GGAATTCCATATGATGAGCATCAGACCAACGACC	This study
pAL-Rv2617c-R	CCATCGATTTAAGGTCTCCCGATGCC	
SigA_F	GTGGCAGCGACAAAGGCAAGCCCGG	This study
SigA_R	CGTCTTTGCGTGCCTGTCG	
SigB_F	GTCTGGTCCGTGGGATGGAGAAGTT	This study
SigB_R	CTCGGCTGTGCTCAAGTAGGTCGTT	
SigE_F	TTCAACGACACTGACTGGGTGGAGC	This study
SigE_R	GTAGGTGCCTGGCTGGTAGTTCTGC	
SigH_F	ACGCCAAGCACTCCTCAACGGGTCT	This study
SigH_R	ACCAAGGTGCGGGACTCGGATGAAC	
MSMEG_3060_F	TTGTGGGGCGCCGCGGCCGTCGTCG	This study
MSMEG_3060_R	CCAGAACCACGCCAACAACG	
MSMEG_3138_F	CAAATCGTTCGCACCGTCGTTCGCC	This study
MSMEG_3138_R	GGTTGGACACCTGCTTGCCCTTCTT	
MSMEG_4264_F	ACCGACCGCATCCTGGC	This study
MSMEG_4264_R	CGCAGCAGCAGTGGGATC	
MSMEG_4903_F	GGCTACCTGTAACCCCAGCAACTCG	This study
MSMEG_4903_R	GCAGCAAATCCACTTCGGTCAGCAC	
MSMEG_6933_F	CGTGTCGGGCGAATAGTCCAATCTC	This study
MSMEG_6933_R	GAAGGTCTGGCGGTAAGTGTAGTCC

### 2.3 Rv2617c does not influence *M. smegmatis* growth kinetics in aerobic conditions

**Fig. 3:**
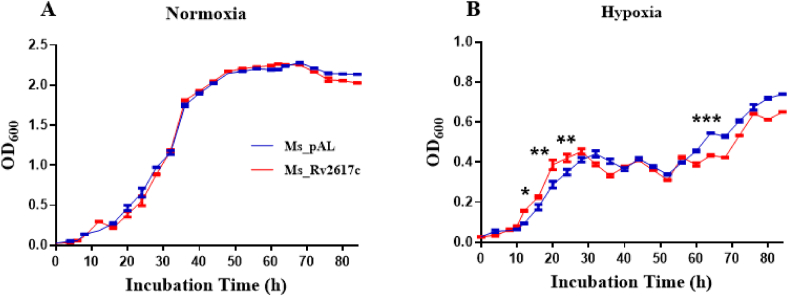
Bacterial growth assessment. (A) Ms_pAL and Ms_Rv2617c were grown in MB 7H9 medium supplemented with 0.05% (v/v) Tween80, 0.5% glycerol, and 1% acetamide. (B) Discoloration of methylene blue (0.2 μg/mL) around 10 h after inoculation of bacteria in flasks sealed with hypoxia plugs indicated the onset of hypoxia. The OD_600_ was determined at an interval of 4 h, and the data are the mean of three replicates. Data are shown as the means ± SD of three independent experiments (*p < 0.05, **p < 0.01, and ***p < 0.001).

Forrellad et al. showed that *M. tuberculosis* Rv2617c mutant was impaired to replicate in the mice lung compared to *M. tuberculosis* wild type [5]; however, Sassetti et al. found that Rv2617c inactivation was advantageous for *M. tuberculosis in vitro* growth [14]. In this study, an investigation of Rv2617c effect on *M. smegmatis* growth under normal conditions (aerobic cultures) during 3 days showed that Rv2617c does not affect *M. smegmatis* growth kinetic (Fig. 3A). Hypoxia is a condition of low or inexistent oxygen tension that *M. tuberculosis*, unfortunately, escapes within host macrophages. We thus explored Rv2617c involvement in this process and found that both strains showed globally reduced growth kinetics in hypoxic conditions compared to normal conditions; however, at the onset of hypoxia (10 h–32 h), Ms_Rv2617c showed significant growth advantage while at the late stage (60 h–80 h), the trend was reversed compared to Ms_pAL (Fig. 3B), suggesting that Rv2617c relatively regulates mycobacterial responses to hypoxia.

### 2.4 Rv2617c enhances *M. smegmatis* survival in the presence of *in vitro* stressors

**Fig. 4:**
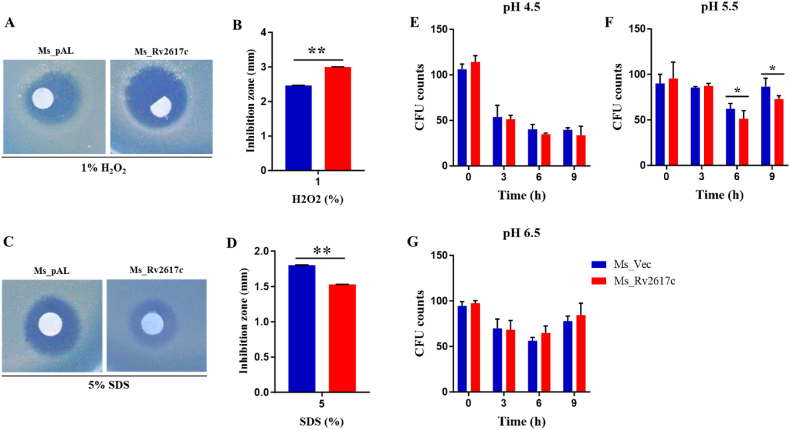
Rv2617c is involved in mycobacterial stress response. (A) Disc diffusion assay of 1% H_2_O_2_ on agar plates with 0.7% agar mixed with Ms_pAL or Ms_Rv2617c, and (B) the diameter of the complete inhibition zone was measured after 3 days of incubation at 37 °C. (C) Disc diffusion assay of 5% SDS on agar plates with 0.7% agar mixed with Ms_pAL or Ms_Rv2617c, and (D) the diameter of the complete inhibition zone was measured after 3 days of incubation at 37 °C. (E), (F), and (G) Ms_pAL and Ms_Rv2617c were exposed to adjusted 7H9 medium pHs ( 4.5, 5.5, and 6.5) for 3 h, 6 h, and 9 h, respectively. Ms_Rv2617c showed significant growth defects, after 6 h and 9 h incubation in pH 5.5-adjusted 7H9 medium compared to Ms_pAL. All cultures have been previously induced with 1% acetamide and data are shown as the means ± SD of 3 independent experiments (*p < 0.05, **p < 0.01, and ***p < 0.001).

The knockout of Rv2617c led to a growth defect in *M. tuberculosis* upon exposure to oxidative stress, indicating that Rv2617c is involved in *M. tuberculosis* resistance to oxidative stress [5]. To confirm that *M. smegmatis* overexpressing Rv2617c also resists the same stress *in vitro*, we assessed the susceptibility of recombinants by spotting 1% H_2_O_2_ on lawns of 0.7% agar mixed with Ms_pAL or Ms_Rv2617c strains. We found that the diameter of the complete inhibition zone of Ms_Rv2617c was bigger than that of Ms_pAL (Fig. 4A and B), suggesting that Ms_Rv2617c might be more susceptible to H_2_O_2_ than Ms_pAL. The same assay was performed with 5% SDS spots; however, Ms_Rv2617c diameter of the complete inhibition zone was significantly smaller than that of Ms_pAL (Fig. 4C and D), indicating that Rv2617c may be involved in mycobacterial SDS stress resistance. One of the harsh barriers faced by *M. tuberculosis* within macrophages is the acidified phagolysosomal environment. We, therefore, mimicked this environment by gradually adjusting 7H9 medium pHs to acidic (pH = 4.5), intermediate (pH = 5.5), and standard (pH = 6.5) ones, respectively. Strains were grown for 9 h, and sample aliquots were collected every 3 h, ten-fold serially diluted, and spotted on 7H10-prefilled plates. The findings demonstrated that at pH 4.5, Ms_Rv2617c and Ms_pAL growth decreased over time with no significant difference between both strains (Fig. 4E). However, at pH 5.5, both strains had comparable increased growth at 0 h and 3 h before observing a reduced Ms_Rv2617c growth at 6 h and 9 h compared to the control (Fig. 4F). In addition, at pH 6.5, Ms_pAL and Ms_Rv2617c showed comparable relative growth over time (Fig. 4G). Altogether, these findings suggest that *in vitro* Rv2617c overexpression relatively regulates Mycobacterial stress responses.

### 2.5 Rv2617c promotes *M. smegmatis* resistance to diamide

**Fig. 5:**
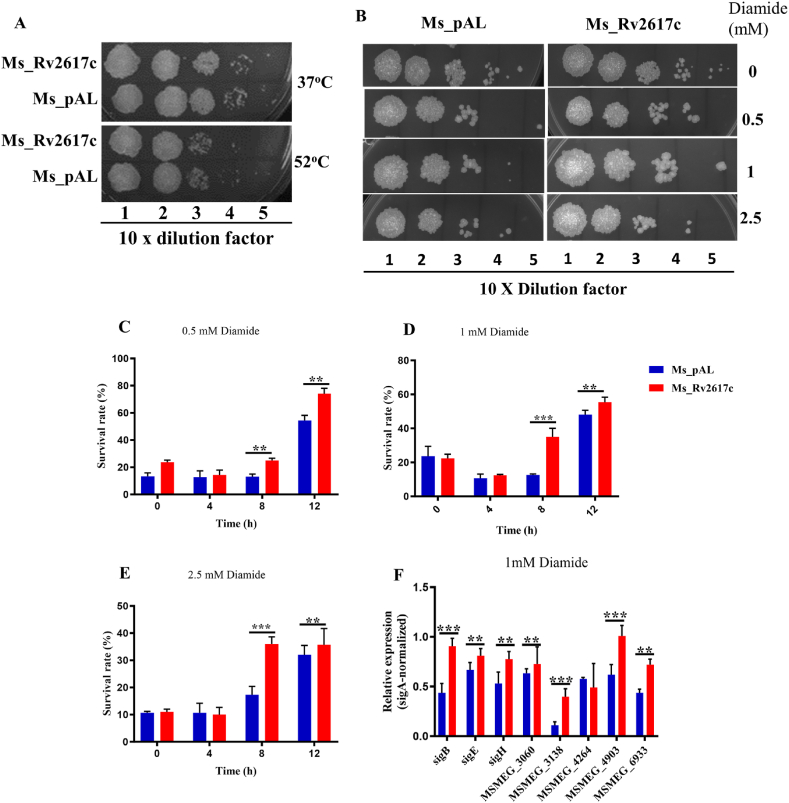
Rv2617c is involved in diamide stress resistance. (A) Survival of Ms_pAL and Ms_Rv2617c after treatment under heat shock conditions. Aliquots from heated (37 °C and 52 °C) samples were 10-fold serially diluted and spotted on 7H10 plates. B) Bacterial samples (OD_600_ = 0.5) were 10-fold serially diluted and spotted on 7H10 plates containing indicated concentrations of diamide. (C), (D), and (E) Ms_pAL and Ms_Rv2617c were exposed to 0.5, 1, and 2.5 mM diamide for 4 h, 8 h, and 12 h, respectively. The results were analyzed after incubation at 37 °C for 3 days. (F) RT-PCR analysis of the expression levels of critical genes of known mycobacterial stress regulators. All cultures have been previously induced with 1% acetamide, and data are shown as the means ± SD of 3 independent experiments (*p < 0.05, **p < 0.01, and ***p < 0.001).

We first subjected both strains to heat shock (37 °C and 52 °C) and found that both strains showed similar colonial growth at 37 °C and 52 °C, with reduced growths at dilutions 10^−4^ and 10^−5^, respectively (Fig. 5A). Then, strains were exposed to the thiol oxidant diamide (0, 0.5, 1, and 2.5 mM) [15], whether on solid agar or liquid medium. Ms_Rv2617c grew more on solid agar after 3 days of incubation compared to Ms_pAL (Fig. 5B). This significant discrepancy was observed in liquid medium as well, precisely at 8 h and 12 h, respectively (Fig. 5C, D, and 5E). To investigate the transcriptional activation response following diamide exposure in both strains, the mRNA expression levels of known sigma factors, such as SigB, SigH, and SigE involved in mycobacterial stress responses, or those of other mycobacterial stress (diamide) drivers (MSMEG_3060, MSMEG_3138, MSMEG_4264, MSMEG_4903, and MSMEG_6933) [16,17] were assessed by qRT-PCR from cDNAs-derived pretreated Ms_Rv2617c and Ms_pAL cultures. Globally, the mRNA expression levels of the aforementioned genes were remarkably upregulated in Ms_Rv2617c than in Ms_pAL (Fig. 5F). Altogether, these findings suggest that Rv2617c induces diamide resistance of *M. smegmatis*.

### 2.6 Rv2617c relatively regulates *M. smegmatis* cell wall permeability

**Fig. 6:**
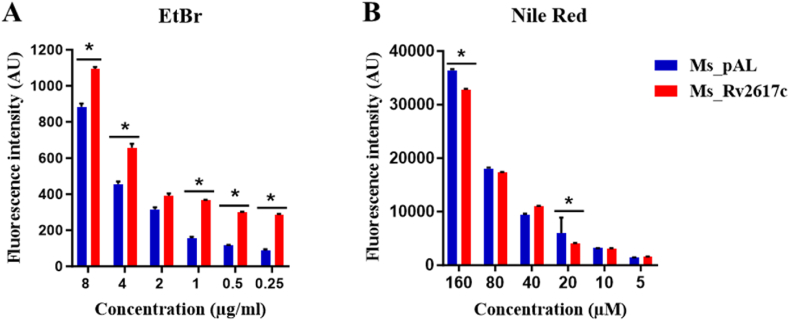
Uptake of decreasing concentrations of EtBr (8–0.25 μg/mL) and Nile Red (160–5 μM). (A) Ms_Rv2617c's accumulation of EtBr is higher than that of Ms_pAL. (B) Ms_Rv2617c's accumulation of Nile Red is lower than that of Ms_pAL. The data reported represent the means ± SD of 2 independent experiments (*p < 0.05, **p < 0.01, and ***p < 0.001).

EtBr is a hydrophilic fluorescent dye that can pass through mycobacterial cell walls and intercalate into DNA and RNA, while Nile Red is a hydrophobic fluorescent dye able to stain intracellular lipid droplets (lipid-rich environment) [18]. As these two dyes are often used to measure mycobacterial membrane permeability, we measured and compared EtBr (0.25, 0.5, 1, 2, 4, and 8 μg/mL) and Nile Red (5, 10, 20, 40, 80, and 160 μM) uptakes by Ms_Rv2617c and Ms_pAL every 5 min during 35 min. The results showed that Ms_Rv2617c significantly accumulated EtBr (Fig. 6A), whereas it showed a significant decrease in Nile Red uptake compared to Ms_pAL (Fig. 6B). Taken together, these results not only indicate a relative regulation of *M. smegmatis* cell wall permeability by Rv2617c but also suggest a modified cell wall lipid content.

### 2.7 Rv2617c is not involved in mycobacterial response to antibiotics

**Fig. 7:**
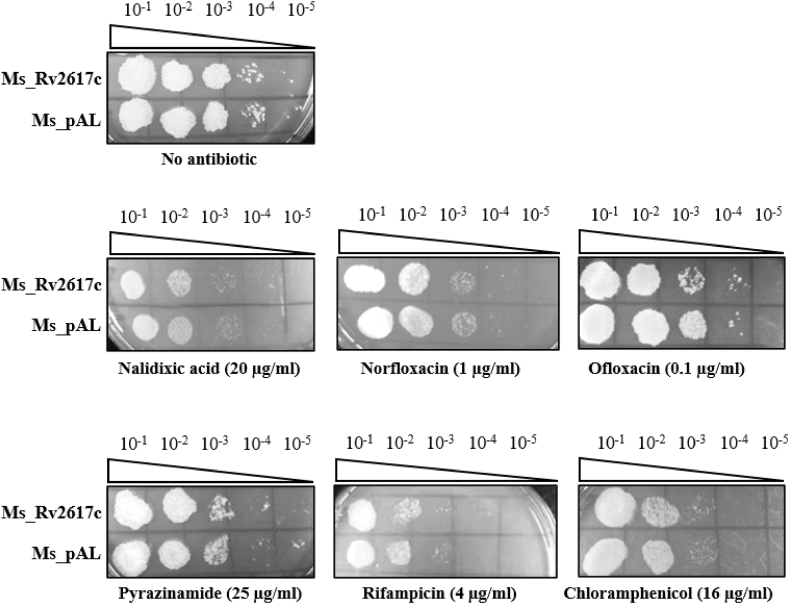
Rv2617c is not involved in antibiotic susceptibility. 10-fold serial dilutions of Ms_pAL and Ms_Rv2617c cultures (OD_600_ = 0.5, ∼4*10^8^ cells/mL) were spotted on MB 7H10 without or with nalidixic acid (20 μg/mL), norfloxacin (1 μg/mL), ofloxacin (0.1 μg/mL), pyrazinamide (25 μg/mL), rifampicin (4 μg/mL), or chloramphenicol (16 μg/mL). The results were recorded after 3 days of incubation at 37 °C.

Consistent with the observed high membrane permeability, we expected a higher bactericidal effect of antimicrobial agents on Ms_Rv2617c strain. Thus, we subjected both strains to anti-TB first- and second-line drugs, including nalidixic acid (20 μg/mL), norfloxacin (1 μg/mL), ofloxacin (0.1 μg/mL), pyrazinamide (25 μg/mL), rifampicin (4 μg/mL), and chloramphenicol (16 μg/mL). Expectedly, we observed a remarkable bactericidal effect of anti-TB drugs on Ms_Rv2617c, although no significant difference between Ms_pAL and Ms_Rv2617c growths was observed (Fig. 7). Rv2617c may not be involved in mycobacterial resistance to antibiotics.

### 2.8 Rv2617c overexpression alters *M. smegmatis* phenotypic properties

**Fig. 8:**
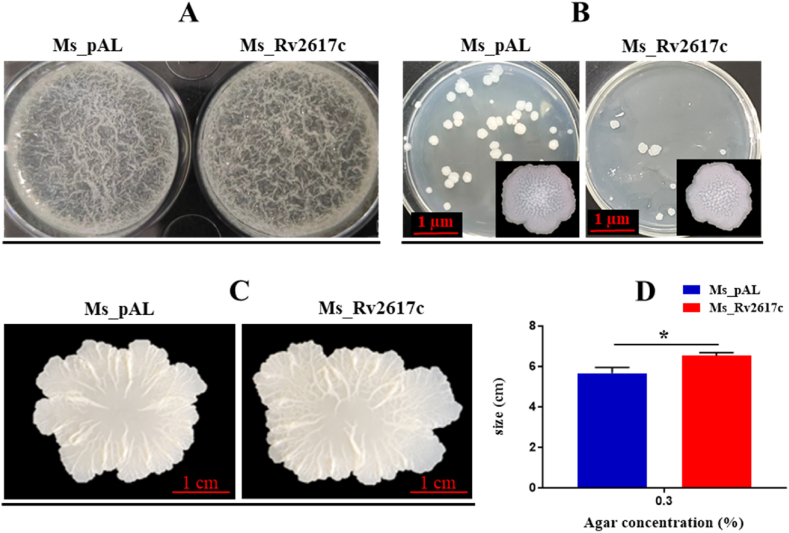
Rv2617c overexpression affects *M. smegmatis* phenotypes. (A) Biofilm formation of Ms_pAL and Ms_Rv2617c in acetamide-induced 7H9 medium. (B) Single-cell colony of strains on MB 7H10 plates. Few Ms_Rv2617c colonies are observed on the plate and show a less compact shape in the center. (C) Sliding motility assay of Ms_pAL and Ms_Rv2617c on 0.3% 7H9 agar. Ms_Rv2617c increases in size. Induced bacterial cultures (OD_600_ = 0.5, ∼4*10^8^) were used for the above assays. (D) Sizes of colonies on the culture dishes were measured after 3 days of incubation at 37 °C. The Figure scale is indicated where required. Data are shown as the means ± SD of 3 independent experiments (*p < 0.05, **p < 0.01, and ***p < 0.001).

The above findings indicate that Rv2617c is involved in mycobacterial stress responses. As modifying cell wall components is a tactic used by bacteria to adapt to stresses [19], we sought to investigate Rv2617c effect on *M. smegmatis* phenotypic properties. Analysis of both strains biofilm showed no difference (Fig. 8A); however, the single clone morphology of Ms_Rv2617c on 7H10 solid agar was less compact in the center with only a few grown colonies on the plate (Fig. 8B) and showed a drastically increased motility on 0.3% agar compared to Ms_pAL (Fig. 8C and D). These findings suggest that Rv2617c may alter *M. smegmatis* cell surface properties.

### 2.9 Rv2617c promotes mycobacterial resistance to phage infections

**Fig. 9:**
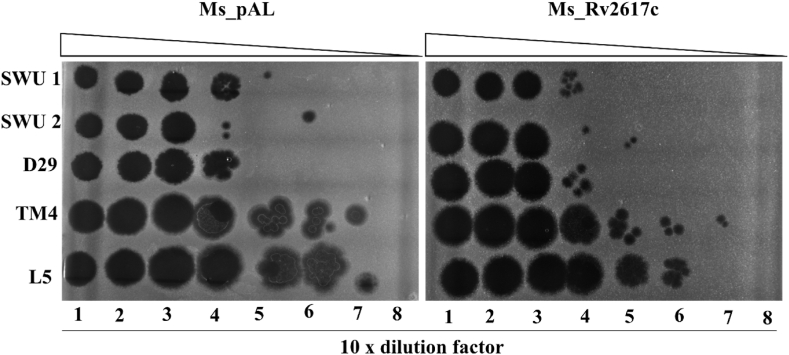
Phage susceptibility of Ms_pAL and Ms_Rv2617c. Spot clearance of lawns of Ms_pAL and Ms_Rv2617c cultures (OD_600_ = 1, ∼8*10^8^ cells/mL) after treatment with 10-fold diluted (10^−1^ to 10^−8^) mycobacteriophages SWU1, SWU2, D29, TM4, and L5. The results were compared after 3 days of incubation at 37 °C.

**Fig. 10:**
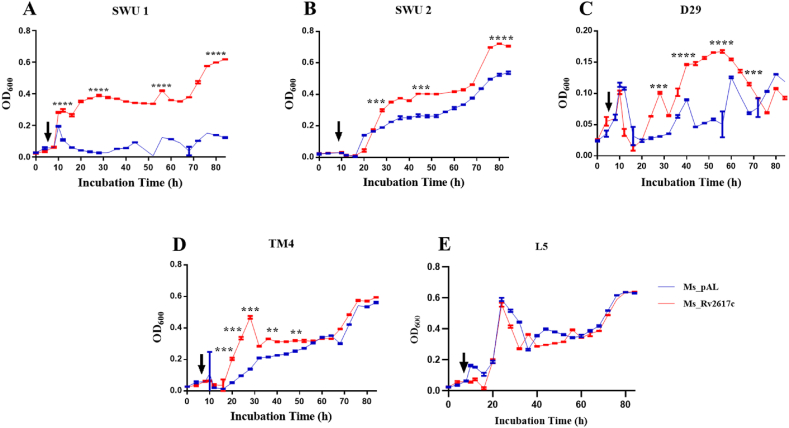
Bacterial growth kinetics upon phage infections. (A), (B), (C), (D), and (E) Ms_pAL and Ms_Rv2617c strains were grown with mycobacteriophages SWU1, SWU2, D29, TM4, and L5, respectively. OD_600_ was measured every 4 h during 88 h. The black arrows indicate mycobacteriophage inoculation time in mycobacterial cultures. Data are shown as the means ± SD of 3 independent experiments (*p < 0.05, **p < 0.01, ***p < 0.001, and ****p < 0.001).

Phage infection steps include adsorption to the host, DNA injection, replication, transcription, translation, progeny phage assembly, host cleavage, and mature phage release [20]. The development of a phage-resistant phenotype can result from host interference at any stage of the life cycle. In this study, mycobacterial (Ms_Rv2617c and Ms_pAL) susceptibility to SWU1, SWU2, D29, TM4, and L5 phages (amplified and purified from *M. smegmatis* mc^2^155 wild-type in our laboratory) was assayed either on solid (titration) or in liquid (MOI of 0.1; using spectrophotometry) conditions. The results demonstrated that Ms_Rv2617c resisted phage infections compared to Ms_pAL on solid agar, especially at the following dilutions: *i*) dilutions 10^−4^ and 10^−5^ for SWU1, *ii*) dilutions 10^−4^ and 10^−6^ for SWU2, *iii*) the dilution 10^−4^ for D29, *iv*) dilutions 10^−5^, 10^−6^, and 10^−7^ for TM4, and *v*) dilutions 10^−5^, 10^−6^, and 10^−7^ for L5 (Fig. 9). Interestingly, the same trend was observed in liquid medium (see Materials and Methods for phage titers, MOI, and cultures OD_600_) where Ms_Rv2617c had a remarkable growth advantage in presence of SWU1, SWU2, D29, and TM4 compared to Ms_pAL although both strains globally showed reduced growth kinetics (<OD_600_ = 0.8) (Fig. 10A–D). However, in the presence of L5, Ms_Rv2617c showed relative growth kinetics with Ms_pAL between 10 h-20 h and 25 h–55 h, respectively (Fig. 10E). The above findings associate Rv2617c with *M. smegmatis* resistance to phage infections.

### 2.10 Rv2617c promotes mycobacterial biofilm formation in the presence of phages

**Fig. 11:**
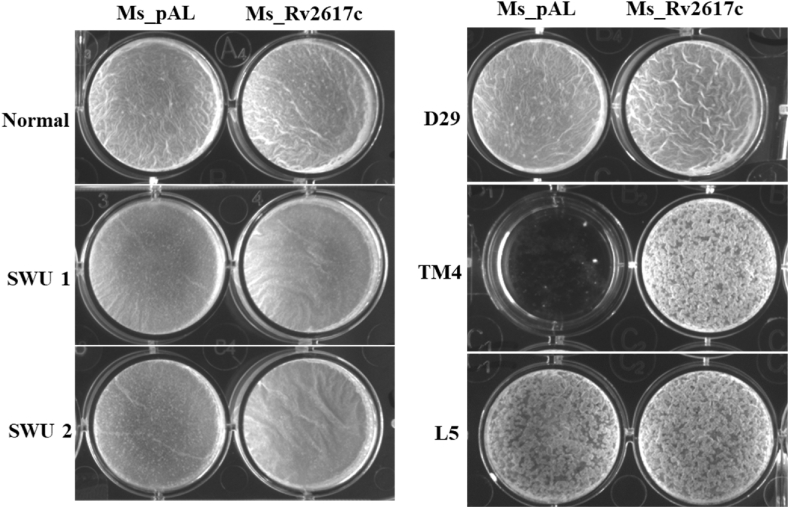
Rv2617c promotes mycobacterial biofilm formation in the presence of phages. Ms_pAL And Ms_Rv2617c strains were cultured in the presence or absence of SWU1, SWU2, D29, TM4, and L5 phages (titers 10^−9^ PFU/mL), respectively. After 3 days of incubation at 37 °C, the results were retrieved and compared.

Phages are reported to release enzymes that destroy mycobacterial biofilms [21]. In this study, we found that Ms_Rv2617c can form biofilm regardless of Ms_pAL (Fig. 8A) and resist phage infections in both solid and liquid conditions (Fig. 9, Fig. 10). We therefore investigated the antibiofilm activity of SWU1, SWU2, D29, TM4, and L5 on Ms_pAL and Ms_Rv2617c biofilms in a 48-well plate filled with 7H9 liquid medium and incubated for 3 days. SWU1- and SWU2-treated Ms_pAL and Ms_Rv2617c cultures could form smooth biofilms as well as D29-treated Ms_pAL and Ms_Rv2617c cultures but with a wrinkled Ms_Rv2617c biofilm. Intriguingly, TM4 infection completely destroyed Ms_pAL biofilm, while that of Ms_Rv2617c was intact with only a few superficial holes. Besides, Ms_pAL and Ms_Rv2617c showed similar biofilms with superficial holes upon L5 infection (Fig. 11). Taken together, these findings indicate the role of Rv2617c in promoting mycobacterial biofilm resistance to TM4 infection.

## 3 Discussion

Macrophage intrinsic barriers, such as acidic environment (low pH), oxygen tension depletion (hypoxia), nutrient starvation, and oxidative stress, are host immune defense mechanisms hostile to intracellular pathogens survival and replication, including *M. tuberculosis*. The latter can evolve escaping strategies, and one of its arsenal weapons is expressing its cell wall-associated virulence determinants. Some *M. tuberculosis* virulence factors have been determined; however, more are still unknown, and the mechanisms of action are far from being fully understood. Accordingly, we functionally characterized *M. tuberculosis* Rv2617c transmembrane protein by exposing *M. smegmatis* expressing Rv2617c (Ms_Rv2617c) to mimicked intracellular macrophage environments and diverse mycobacteriophages.

In this study, phylogenic analysis and MSA showed that Rv2617c is conserved among pathogenic and non-pathogenic mycobacteria species and belongs to the DoxX domain family proteins. This suggests that Rv2617c has been conserved during those species evolution, and may have different functions in the latter.

Besides, we found that Rv2617c can be successfully expressed in *M. smegmatis* and does not affect host growth kinetic in normal conditions. However, in the presence of mimicked *in vitro* conditions, such as hypoxia, SDS, intermediate pH (5.5), and diamide, Ms_Rv2617c showed a relative growth advantage, indicative of Rv2617c involvement in survival and stress responses of *M. tuberculosis* within macrophages. Inversely, Ms_Rv2617c showed growth defects when exposed to oxidative stress (H_2_O_2_). This finding is in disagreement with previous studies that explored Rv2617c function in the same stress condition, reporting that Rv2617c and P36 act in concert to prevent bacterial (*M. tuberculosis* and *M. bovis*) damage upon hydrogen peroxide treatment [5]. We speculated that this discrepancy might be associated with the absence of P36 (Erp) ortholog in *M. smegmatis*.

Next, we found that Rv2617c had no impact on mycobacterial biofilm but significantly affected mycobacterial motility, single-cell morphology, and cell wall permeability/porosity. Since the mobility, biofilm, and cell wall permeability/porosity can be associated with changes in the lipid composition of the mycobacterial cell membrane and cell wall [22], it suggests that Rv2617c protein expression may influence the lipid composition of *M. smegmatis* by regulating the genes involved in cell wall lipid biosynthesis. Profiling both Ms_Rv2617c and Ms_pAL lipid compositions would inevitably bring more insights for the understanding of the mechanism of action of the Rv2617c protein.

Mycobacteriophages are viruses that infect mycobacteria, and mycobacteriophage-mediated mycobacterial growth inhibition represents a potentially effective alternative to antibiotic resistance [[23], [24], [25]]. Mycobacteriophages SWU1, SWU2, D29, TM4, and L5 can infect *M. smegmatis* either in a lytic or lysogenic way by affecting the host cell wall contents (lipids, peptidoglycans), with an impact on the host phenotype and behavior in response to stresses [9,11,[26], [27], [28], [29], [30], [31]]. Rv2617c promoted mycobacterial resistance against all mycobacteriophages mentioned above (excepted L5: no significant difference) in both soft agar plates and liquid broth medium, and intriguingly, Ms_Rv2617c biofilm formation was not suppressed by TM4 infection. These findings suggest another role of Rv2617c in mycobacterium-mycobacteriophage crosstalk and open the avenue of considering Rv2617c as a potential target for mycobacteriophage-based TB therapy.

Altogether, this study reports on the roles of Rv2617c and how it may contribute to *M. tuberculosis* pathogenicity within its human host. Besides, the strongest involvement of the Rv2617c protein in mycobacterial resistance to mycobacteriophage infections opens new avenues for further research. Finally, the global transcriptional responses or lipid profiles between both strains under study as well as the exploration of *M. smegmatis* expressing Rv2617c behavior within human macrophages or animal models (mice) have not been explored and thus limit the current work in this way. These aspects would have provided more insights (*in vitro* transcriptional responses and *in vivo* survival, cytokine secretion, induced signalling pathways as well as activated cell deaths within macrophages) to Rv2617c functional mechanisms. These aspects may represent exciting perspectives for future research directions.

## 4 Materials and Methods

### 4.1 Bioinformatics

The Rv2617c protein sequence was downloaded from Mycobrowser (https://mycobrowser.epfl.ch/). Phylogenetic tree and MSA were performed as previously described by Li et al. [32]. Briefly, after Rv2617c protein sequence retrieval, the latter was blasted in blast protein (Blastp) command (https://blast.ncbi.nlm.nih.gov/Blast.cgi); mycobacterial organisms sharing Rv2617c homologous proteins were then used for phylogenetic tree building by using MEGA 11. MSA was built with three closely related mycobacterial species using Esprit 3 online software.

### 4.2 Bacterial strains, plasmids, and growth conditions

For liquid culture, *M. smegmatis* mc^2^155 (Invitrogen) strains were grown in Middlebrook (MB) 7H9 medium supplemented with 0.05% (v/v) Tween80 and 0.5% (v/v) glycerol or in liquid Luria-Bertani (LB) for *E. coli* strains. For solid culture, MB 7H9 supplemented with agar or 7H10 supplemented with 0.5% glycerol was used for *M. smegmatis*, whereas solid LB was used for *E. coli*. Antibiotics were added at the following concentrations: 100 μg/mL ampicillin and 100 μg/mL hygromycin (*M. smegmatis* strains) and 50 μg/mL kanamycin and 100 μg/mL hygromycin (*E. coli* strains). All cultures were incubated at 37 °C. Bacterial strains, plasmids, and primer sequences used in the construction of recombinant strains are listed in Table 1.

### 4.3 Rv2617c's cloning and protein expression

The mycobacterial expression vector pALACE used in this study has been described previously

[33]. The *M. tuberculosis* open reading frame (ORF) Rv2617c was PCR-amplified from the *M. tuberculosis* H37Rv genome using both forward (5′-GGAATTCCATATGATGAGCATCAGACCAACGACC-3′) and reverse (5′-CCATCGATTTAAGGTCTCCCGATGCC-3′) primers, harboring *NdeⅠ* and *ClaⅠ* restriction sites (underlined), respectively. Rv2617c DNA products and the mycobacterial expression vector pALACE were digested separately before being ligated (pAL-Rv2617c) and subsequently introduced into *E. coli* DH5α and *M. smegmatis* by heat shock and electroporation, respectively. Selection of positive clones was carried out on LB agar (*E. coli*) or MB 7H10 agar (*M. smegmatis*) with appropriate antibiotics (see Section 4.2) and confirmed by PCR on colonies.

For Rv2617c expression, recombinant *M. smegmatis* strains (Ms_Rv2617c and Ms_pAL) were cultured in MB 7H9 broth medium (with 50 μg/ml Hyg and 1% acetamide added) until OD6_00_ reached 0.8–1, then bacteria were harvested by centrifugation (8000 × *g*/10 min/4 °C), sonicated (cold 1 × PBS with protease inhibitor P-8849 added), and whole cell lysates were segregated using SDS-PAGE and Western blotting. Recombinant *M. smegmatis* were stored in the refrigerator at −70 °C for further use.

### 4.4 Sliding and single colony morphology

Sliding motility and colony morphology assays were performed as described by Zhao et al. [34], with minor modifications. Briefly, Ms_pAL and Ms_Rv2617c strains were grown into MB 7H9 liquid medium containing 0.05% tween80, 0.33% D-glucose, appropriate antibiotics, followed with acetamide induction until OD_600_ reaches 0.8–1.0. Then, collected bacteria were washed twice with 1 x PBS and resuspended with fresh 7H9 medium to an OD_600_ of 0.5. For sliding, 10 μL of samples was dropped to the center of the 7H9 medium plates filled with 0.3% agar. For single colony morphology, samples were serially diluted (10^−1^ to 10^−5^), and 100 μL of the 10^−5^ dilution was plated on 7H10 solid plates. All samples were incubated at 37 °C for 3 days.

### 4.5 Biofilm formation

Ms_pAL and Ms_Rv2617c were cultured similarly as for sliding and single colony morphology assays but to a final OD_600_ of 0.8 after equilibrating with fresh 7H9 medium. Samples were then added into each well of the 6-well polystyrene plate (60 × 15 mm^2^) culture dishes containing 7H9 medium, acetamide, and 33% D-glucose. The plates were then incubated at 37 °C for 3–5 days with no shaking. The surface growth layers of strains were then compared.

### 4.6 *In vitro* stress assays

These experiments were performed as previously indicated by Huang et al. [35], with minor modifications. For H_2_O_2_ and SDS sensitivity assays, aliquots of Ms_pAL and Ms_Rv2617c cultures (OD_600_ = 0.8) were mixed with 0.7% agar medium and plated onto 7H9 agar plates. Paper spots were then put onto the surface of agar with 10 μL H_2_O_2_ (0.5, 1, and 2%) or SDS (1.25, 2.5, and 5%) added. After incubation at 37 °C for 3 days, the inhibition zones (diameters) caused by the above agents were measured.

For low pH susceptibility, *M. smegmatis* strains were grown to the mid-log phase. Bacterial pellets were collected, washed twice with 1 x PBS, resuspended with fresh 7H9 medium, and the OD_600_ was lastly adjusted to 0.5. Then, 1% of each of the above cultures was inoculated into 10 mL 7H9 broth and grew for 9 h at 37 °C with shaking. Thereafter, sample aliquots were collected every 3 h, 10-fold serially diluted with 1 x PBS, and plated onto MB 7H10-prefilled plates, which were incubated for 3 days at 37 °C. Finally, Cell viability was determined by colony-forming units (CFUs).

### 4.7 Drug susceptibility assay

Firstly, the MICs of antibiotics were determined as previously described [36] using the double broth dilution method. After incubation for 3 days at 37 °C, the lowest concentration that prevented visible growth was defined as the MIC. For bacterial survival within antibiotics, acetamide-induced Ms_pAL and Ms_Rv2617c strains were cultured to late logarithm, then OD_600_ adjusted to 0.8 or 0.5 and 10-fold serially diluted. For susceptibility on solid broth, bacteria (OD_600_ = 0.8) were spotted on 7H9 agar containing 40 x MIC of each antimicrobial drug used in this study. For liquid assessment, Ms_pAL and Ms_Rv2617c strains (OD_600_ = 0.5) were treated with 8 x MIC of different antimicrobial agents at different intervals, then ten-fold serially diluted, and spotted on MB 7H10. The results were recorded after 3 days of incubation at 37 °C, and the CFUs were calculated until the growth of the colony was observed. Assays were performed in triplicates.

### 4.8 Mycobacterial cell wall porosity assessment

Membrane porosity of Ms_pAL and Ms_Rv2617c is assessed by monitoring the cell wall uptake and efflux of ethidium bromide (EtBr) and Nile Red reagents, as described by [26]. Briefly, bacteria were grown in 7H9 in the presence of appropriate antibiotics and 0.05% (v/v) Tween80. When OD_600_ reached 0.4, induction with acetamide was performed, and cultures further grew until OD_600_ reached 1.0. Then, bacteria were collected by centrifugation (8000×*g*/10 min/4 °C), washed twice with 1 x PBS, and resuspended with 0.05% PBST (add 100 or 200 μL of 0.05% Tween80 to 200 mL 1 x PBS) and equilibrated to an OD_600_ of 0.5. After pouring 1 mL of bacteria in 1.5 Ep. tubes, add 10 μL of each dye respectively, homogenize by pipetting, and quickly pour 200 μL of each mixture into the 96-well fluoroplastic. Dye uptakes assessment was made during 60 min (5 min for each time point measurement) with the following parameters: Nile Red (excitation: 540 nm and emission: 630 nm) and EtBr (excitation: 545 nm and emission: 600 nm). The resulting data were extracted and analyzed with GraphPad software.

### 4.9 Mycobacteriophage assays

All assays related to phage infections of *M. smegmatis* strains in this study followed the methods indicated by Fan et al., 2015 [8].

#### 4.9.1 Phage purification

100 μL of phage lysates were mixed with 1 mL of late-log phase *M. smegmatis* mc^2^155 cells and 10 mL of soft agar (0.7% agar, 1 mM CaCl_2_). This mixture was poured on a 150 mm plate containing 7H9 agar (7H9 broth base + 2% agar). Eighteen such plates were prepared. Allow plates to harden, and then incubate at 30 °C overnight. Add 10 mL MP buffer to each plate. Following overnight incubation at 4 °C, the buffer was pipetted off the plate. Centrifuge 3500*g* for 5 min at 4 °C to pellet bacterial cells. Transfer the supernatant to a 250 mL flat-bottom plastic bottle. Add 10% polyethylene glycol (PEG 4000) and NaCl to 1 M. Stir this at 4 °C overnight. Centrifuge the precipitated phage for 5 min 3500 g at 4 °C. Resuspend the phage pellets in 6 mL MP buffer. Pipette gently up and down, and rock at 4 °C overnight. Centrifuge at 3500 g at 40 °C, and then save the clear supernatant. The phage was purified using CsCl density gradient centrifugation with d = 1.45 upper and d = 1.50 lower CsCl layers. The phage band at the interface between the two CsCl layers was removed with a syringe and needle.

#### 4.9.2 Plate morphology

Two distinct adjacent plaques were chosen for further morphology characterization. The parameters for the clear/turbid nature and appearance of the plaques were noted. 500 μL of MP buffer, 500 μL of *M. smegmatis* mc^2^155 suspension (without Tween80 and OD_600_ = 1), and 4.5 mL of soft agar (0.7% agar and 1 mM CaCl_2_) were mixed and poured on a 100 mm plate containing 7H10 agar. 5 μL of SWU1, SWU2, D29, TM4, and L5 lysates (serial dilutions from 10^−1^ to 10^−8^ PFU/mL) were spotted on plates after the top agar was cooled and hardened completely. After the spots evaporated, plates were incubated overnight at 37 °C, and the sizes of halos surrounding the plaques were measured.

#### **4.9.3** Phage infection of M. smegmatis in liquid medium

200 μL of cultures were added to 5 mL 7H9 broth supplemented with OADC and incubated overnight until OD_600_ reached 1.5 (no Tween80 added). Gently, cells were resuspended and OD_600_ adjusted to 0.1 with prewarmed 7H9 broth supplemented with ADC (9/l, v/v). Then, CaCl_2_ was added to the culture (OD_600_ = 0.1) to a final concentration of 1 mM. Thereafter the mycobacteriophage is added and the whole is incubated at 37 °C for 15 min before placing the culture on a shaker. A culture of mycobacterial cells at an OD_600_ of 0.1 contains approximately 10^−7^ cells per mL. Cells are next infected with a MOI of 0. 1 and 10^−9^ PFU/mL. Finally, OD_600_ is measured every 3 h until OD_600_ became constant.

### 4.10 qRT-PCR validation

Ms_pAL and Ms_Rv2617c strains were cultured in the presence of appropriate concentrations of diamide to an OD_600_ of 1.0. Total RNAs were extracted from Ms_pAL and Ms_Rv2617c strains following the manufacturer instructions (TIANGEN, China). cDNA was synthesized using random hexamer primers following the manufacturer instructions (TIANGEN, China). Expressions of the critical genes regulated by Rv2617c following bacterial exposure to diamide stress were detected by qRT-PCR amplification performed in Bio-Rad IQ5 using the following PCR parameters: 95 °C for 5 min, and 40 cycles at 95 °C for 30 s, 60 °C for 30 s and 72 °C for 30 s. Then, using “normalized expression (ΔΔCq)” (Bio-Rad CFX manager 3.1), all gene expressions were standardized to that of the internal control SigA (MSMEG 2758) [37,38]. The primers used for this assay are listed in Table 1.

### 4.11 Statistical analysis

All experiments were performed in triplicate, and data analysis was performed using Excel 2019 and GraphPad Prism 7. All data are presented as means ± standard deviation (SD). *P*-value (statistical significance) was decided based on the student *t*-test and one-way ANOVA. *P*-value was considered significant (*p < 0.05, **p < 0.01, ***p < 0.001, ****p < 0.0001).

## Funding

This work was supported by the 10.13039/501100010890Chinese Government Scholarship (CSC No. 2019GBJ002789) and the 10.13039/501100001809National Natural Science Foundation (grant numbers: 82072246, 81871182 and 81371851).

## Data availability statement

All data generated in this study are included in this article.

## CRediT authorship contribution statement

**Liadrine Moukendza Koundi:** Writing – review & editing, Writing – original draft, Project administration, Investigation, Formal analysis, Conceptualization. **Ulrich Aymard Ekomi Moure:** Writing – review & editing, Investigation, Conceptualization. **Funmilayo Grâce Boni:** Visualization, Validation. **Insaf Hamdi:** Visualization, Validation. **Lin Fan:** Visualization, Validation. **Jianping Xie:** Visualization, Validation, Supervision.

## Declaration of competing interest

The authors declare that they have no known competing financial interests or personal relationships that could have appeared to influence the work reported in this paper.
