# Supplementary material for: Mycobacterium tuberculosis Rv2617c is involved in stress response and phage infection resistance
Source: Heliyon. 2024 Mar 5;10(5):e27400. doi: 10.1016/j.heliyon.2024.e27400 (PMC10943396; doi:10.1016/j.heliyon.2024.e27400)

**Additional file: uncropped/full images of cropped images cited in the main manuscript**

**Title: *Mycobacterium tuberculosis* Rv2617c is involved in stress response and phage infection resistance.**

Liadrine Moukendza Koundi^1^*, Ulrich Aymard Ekomi Moure^2,3^, Funmilayo Grâce Boni^1^, Insaf Hamdi^1^, Lin Fan^4^, Jianping Xie^1^*

^1^Institute of Modern Biopharmaceuticals, State Key Laboratory Breeding Base of Eco-Environment and Bio-Resource of the Three Gorges Area, Key Laboratory of Eco-environments in Three Gorges Reservoir Region, Ministry of Education, School of Life Sciences, Southwest University, Chongqing, China.

^2^ The Ninth People’s Hospital of Chongqing, Affiliated Hospital of Southwest University, Chongqing, China.

^3^Cancer Center, Medical Research Institute, Southwest University, 400716 Chongqing, China.

^4^Shanghai Clinic and Research Center of Tuberculosis, Shanghai Pulmonary Hospital, Tongji University School of Medicine, Shanghai Key Laboratory of Tuberculosis, Shanghai, China

*Corresponding authors:

Liadrine Moukendza Koundi, [liadrinekoundi@gmail.com](mailto:liadrinekoundi@gmail.com); Pr. Jianping Xie, [georgex@swu.edu.cn](mailto:georgex@swu.edu.cn);

**Fig. 2A. PCR-amplification of Rv2617c gene (441 bp).** The Rv2617c gene amplification is shown on the right side of the picture.


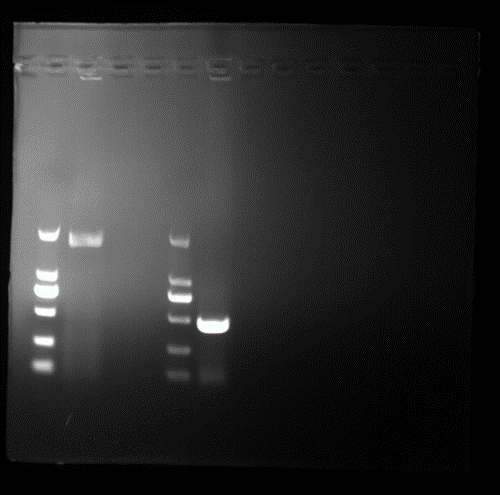


**Fig. 2B. PCR-amplification of Rv2617c gene (441 bp) from Ms_Rv2617c strain.** The Rv2617c gene amplification from bacterial colonies is depicted on the right side of the picture.


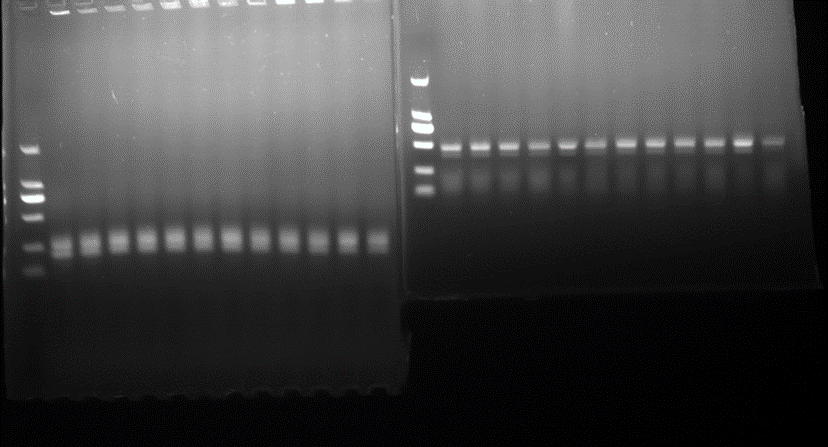


**Fig. 2C. SDS-PAGE analysis of Rv2617c protein expression from Ms_Rv2617c culture lysates (associated with His-tag protein: 16.2 kDa).**

Gel description (from left to right): Wells 6 (marker), 7-8 (Ms_Rv2617c samples: total, supernatant, and sediment proteins, respectively), and 9 (Ms_pAL sample: total protein).


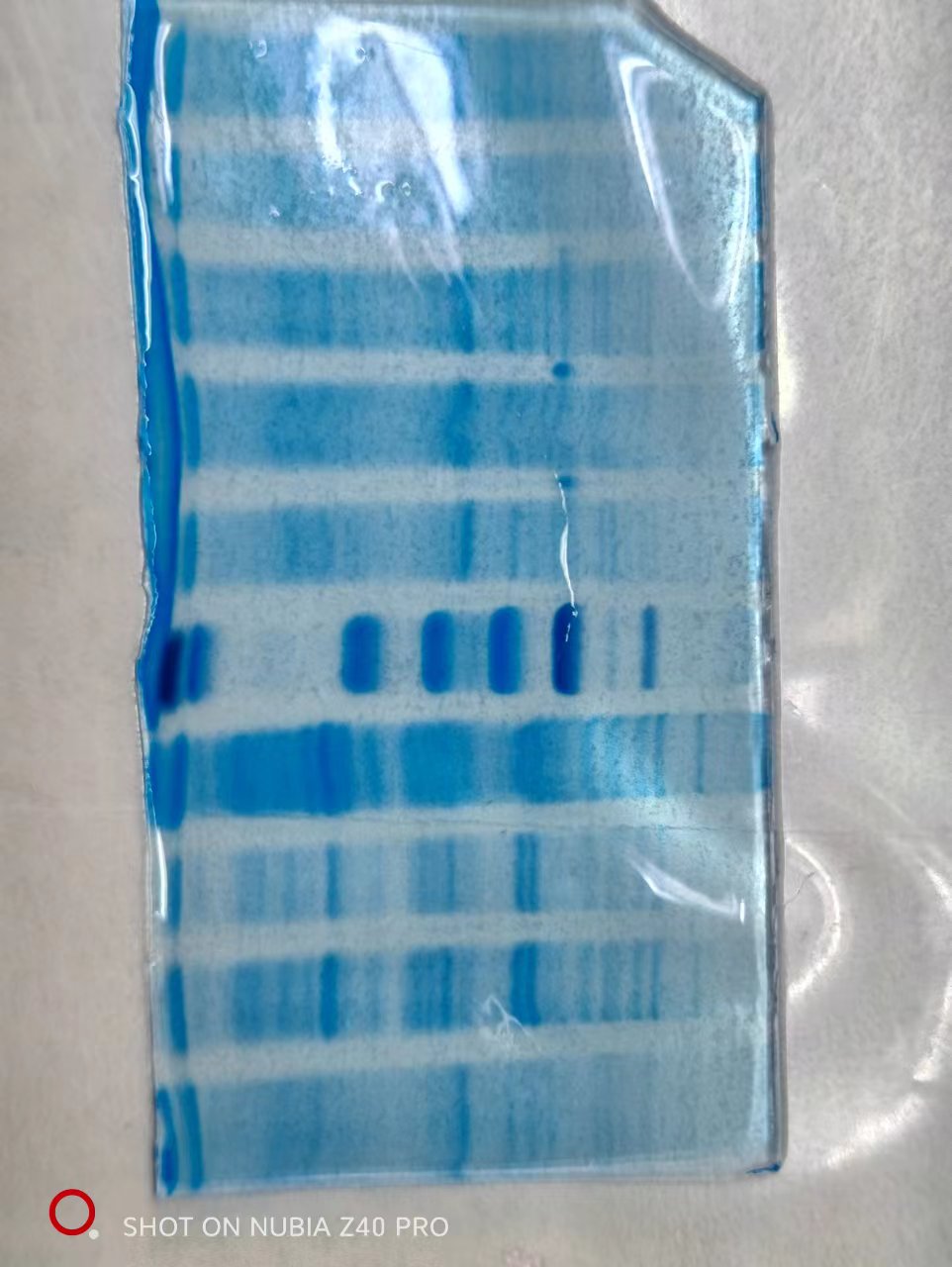


**Fig. 2D. Western Blot analysis of Rv2617c protein expression from Ms_Rv2617c culture lysates (associated with His-tag protein: 16.2 kDa).**

Gel description, second membrane on the right side (from left to right): Wells 1 (marker), 2 (Ms_pAL sample: supernatant), 3 (Ms_Rv2617c supernatant: sample selected), 4 (Ms_pAL sample: supernatant), and 5 (Ms_Rv2617c supernatant: sample not selected).


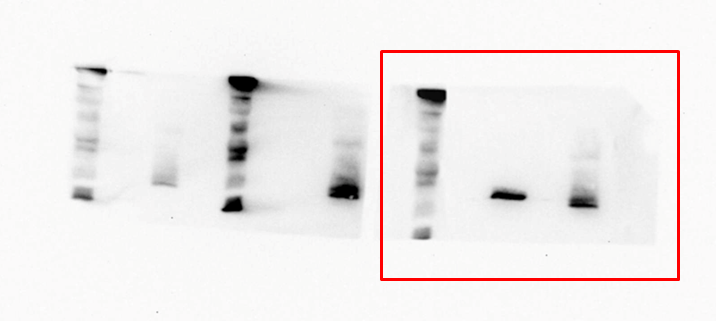

Supplement: Multimedia component 1 [file mmc1.docx]
